# Pennsylvania

**Published:** 1899-05

**Authors:** H. B. Felton

**Affiliations:** Chairman


Secretary.

### PENNSYLVANIA STATE VETERINARY MEDICAL ASSOCIATION.

*Report of Committee on Intelligence and Education.*[1]

In reviewing the events of the past year we find that much has occurred of vital interest to the veterinarian that may properly be classed under the head of intelligence and education. From such a wealth of material it is hard to make a comprehensive report which shall be included within the limits to which we are confined.

In regard to the practice of veterinary medicine, we believe the outlook

[1] Read before the annual meeting, March 7, 1899.

is very encouraging. With a revival of business we are beginning to see a return of old-time prosperity. Bicycling as a fad is becoming a thing of the past, and many are returning to their first love—the horse. The elite have discovered that as a liver regulator and cure for dyspepsia a live horse is better than a dead wheel, while those blessed with a superabundance of adipose tissue, who erstwhile made spectacles of themselves in biking costume astride the festive wheel, have returned to the more sedate pleasure of an airing behind their tried and trusty four-footed steeds. The horse-market at the present time is in a very satisfactory condition. Since the beginning of the year there has been a steady appreciation in the value of horses, with a demand for animals of high quality which could not be fully met. This demand, with the growing export-trade in horses, which is rapidly becoming the most profitable market for our dealers, has encouraged the farmers to again enter the breeding ranks, which should never have been abandoned. But the valuable lesson has been learned that to breed for profit none but the best will do, and the breeder who produces this kind will always find a ready sale with the profit on the right side.

It is true that we are now threatened with the automobile, as we have been with the bicycle and the trolley car, and many of us view with apprehension the formation of companies with millions of capital, which are preparing to furnish vehicles driven by electricity and compressed air for all purposes of business and pleasure. Already we see them flitting through the streets of our large cities, and we wonder what the outcome is going to be. They have not yet been made with the guarantee that they will be blizzard-proof and mud-conquering, but who can doubt that the genius that created them will be able to overcome these obstacles. With the multiplication of good roads, which the autumobile along with the bicycle is bound to create, we believe it is destined to have a large use in the next few years, and to curtail considerably the field of operations of the veterinarian.

Fortunately, however, with our advancing civilization new fields are opening up for the exercise of our profession, and we are encouraged to think that when the deluge shall overtake us we shall still, like Noah, have an ark in which to float. The extension of our possessions to the islands of the sea may furnish a profitable field for the veterinarian. While present conditions are not very encouraging, yet we believe that when American enterprise has become fully established the veterinarian will find profitable employment. Conditions are extremely favorable in certain parts of Cuba, Porto Rico, and Hawaii for raising cattle and carrying on dairy operations. Already a large number of horses and mules have been exported to Cuba, and many more will be needed for the prosecution of enterprises already established.

A bill has been recently introduced into the Legislature of Pennsylvania by Representative Stulb, of Philadelphia, for regulating the milk-supply of the cities and rendering the conviction of offenders against the law less difficult. We do not know the merits of the bill, not having seen a copy of it, but from what we can learn there does not seem to be any probability of the measure passing, as the sentiment of the dealers and the farmers is opposed to it. Among the milk-inspctors of the City of Philadelphia there are three veterinarians, graduates of the University of Pennsylvania. With one exception they have never furnished our Association with any information bearing upon the inspection of milk or the number of diseased cattle found in inspecting dairies. Possibly they have never received a specific invitation to do this, and if this is the case, we hope the invitation will be forthcoming, as they ought to be able to tell us much that would be of interest and profit.

Representative Adams, of Philadelphia, has within a few days also introduced a bill into the Legislature which is said to have the Drovers' Association of West Philadelphia back of it. This bill provides that within thirty days of the passage of the act the Secretary of the Pure-Food Commission of this Commonwealth shall appoint an inspector of meat in each county of the Commonwealth in which is located one or more cold-storage warehouses, for the purpose of inspecting all meats killed outside of the limits of the State. These inspectors shall be graduates of some veterinary college and shall be appointed for the term of four years. It shall be the duty of the meat-inspectors to thoroughly inspect all meat killed outside of the Commonwealth before it is offered for sale, and each carcass shall be marked in such a manner as may be determined by the Secretary of the Pure-Food Commission, and so labelled when offered for sale to the public. Should the meat-inspectors find any meat infected with tuberculosis or any other disease, or determine that any meat has been treated with any preservative drug or material in his judgment deleterious to good health, he shall condemn it, and it shall be unlawful to sell or offer for sale any part of such meat. The pay of inspectors shall be one dollar for each carcass inspected. For the purpose of inspection they are empowered to enter the premises of every firm, corporation, or person who may have meat killed outside the State. The selling of such meat without bearing the mark of the inspection shall be a misdemeanor punishable by a fine of from twenty-five dollars to one hundred dollars, or imprisonment not exceeding sixty days. The provisions made in this bill for the inspection of meat brought into the State are certainly excellent. Its evident purpose is to place such a burden upon Western refrigerated beef as to eventually drive it out of the markets of our State. Whether it would accomplish this result or not, we do not know, but we believe something ought to be done to check the ever-growing Western monopoly which is gradually stifling our home industries, and city-dressed meats, although still extensively advertised by our butchers, and always in demand, are rapidly becoming a fiction. On account of this monopoly the North Philadelphia Drove Yard has closed up, and the Gray's Ferry Abattoir has ceased slaughtering cattle for beef. President Wise, of the North Philadelphia Drove Yard, estimates that less than 1 per cent. of Philadelphia butchers now slaughter their own cattle.

Dr. Hoskins has recently raised quite a breeze among the local dealers by an address delivered before the "Women's Health Protective Association of Philadelphia," in which he charged that there is an utterly inadequate inspection of city dressed meats, and that the slaughter-houses are surrounded with every conceivable kind of dirt and filth, and meat is exposed for sale and carted through the streets of the city without proper protection from germs and dirt. While his charges are probably too sweeping in character, yet we are sorry to say there is too much truth in them, and we believe the plan advocated by Dr. Hoskins, of the establishment of central abattoirs under municipal control, where cattle shall be kept and slaughtered under the best possible sanitary conditions, and every carcass rigidly inspected, to be the best possible solution of the question.

The subject of tuberculosis and its prevention is being brought more prominently before the people than ever before, and veterinarians are enlisting the coöperation of the medical profession in their endeavors to stamp out the disease among cattle. In a report recently made by Dr. Guerard, of New York, to the Senate Commission on Tuberculosis of that State, which is a very valuable contribution upon the subject and ought to be read by every veterinarian, the Doctor says in regard to prevention: "The public, hence, with a care that falls into no mistakes and makes no slips, needs to be protected from diseased meats, and especially from even the very suspicion of an infected milk-supply."

We note with pleasure the improvement in the JOURNAL OF COMPARATIVE MEDICINE AND VETERINARY ARCHIVES by the establishment of the departments of "Therapeutics" and "Surgery." The veterinarian who does not subscribe for this wide-awake journal sustains a loss which can only be computed by rule of compound interest.

We call the attention of the Association to Merck's 1899 *Manual of Materia Medica*, together with a summary of "Therapeutic Indications and Classification of Medicaments." This valuable work will give the veterinarian a concise knowledge of all the recent drugs as well as the older ones, and is compact enough to be carried in the pocket for ready reference. We also call attention to the *Ephemeris*, issued from time to time by E. R. Squibb & Sons, which gives a list and description of the newer remedies which have been tried and proved to be of value.

Legislation for the regulation of the practice of veterinary medicine in the various States seems to be progressing favorably, and Michigan, Illinois, Maine, New Hampshire, and Tennessee will shortly join the ranks of those who have already enacted laws.

We are sorry to have to record the failure of the efforts made to give the veterinarian his proper rank in the army by a proviso inserted in the "Army Reorganization Bill." In the closing hours of Congress the "Hull Bill," which contained this proviso, was ruthlessly set aside and the German "Cockrell Bill" passed instead, which leaves the standing of the veterinarian as it was before. It reminds us of a story told of a young tailor named Berry, who, succeeding to his father's business, once sent in his account to Charles Matthews somewhat ahead of time. Whereupon Matthews, with virtuous rage, wrote him the following note: "You must be a goose—Berry, to send me your bill—Berry, before it is due—Berry. Your father, the elder Berry, would have had more sense. You may look very black—Berry, and feel very blue—Berry; but I don't care a straw—Berry, for you and your bill—Berry." And so Congress says to us: "You are goose—Berries for sending in your bill before its time, and you may look very black—Berries and feel very blue—Berries, but we don't care a straw—Berry for you or your bill." But we serve Congress notice that we shall continue to be rasp—Berries until we accomplish our purpose, and our motto shall be "keeping everlastingly at it brings success." We call the attention of the Association to a pamphlet published by the American Veterinary Association, which explains fully what position the veterinarian is expected to occupy in the army, and the reasons why he should do so. A perusal of this pamphlet will enable every veterinarian to inform himself upon this subject and write intelligently to members of Congress.

The army beef scandal smells up to heaven. The weight of the evidence proves that much of the beef was bad and totally unfit for food. The dirty hand of politics can be traced from the highest official down through the ranks until we come to inspectors appointed with no knowledge of or fitness for the position, but solely on account of political pull—inspectors who did not inspect or whose knowledge of beef-products was as hazy as Schuylkill water. Had the veterinarian held his proper rank in the army and received the proper recognition in the appointment of inspectors, we believe this scandal would have been avoided, as well as the added scandal revealed in "Army Drift" in the JOURNAL of November last, caused by the appointment of men as army veterinarians totally lacking in knowledge and fitness for the position. The "Beef Trust" is howling and lamenting that its export trade will be ruined if the scandal is not hushed up. It has sown to the wind, and it must reap the whirlwind. Through deep humiliation it will probably learn that it pays to be honest. It ought to have learned a lesson from the shippers who several years ago shipped diseased meat to Europe and failed of being sent to the penitentiary, where they belonged; but the Kansas hog has not yet recovered from the stain on his reputation, although the United States has spent millions of dollars to prove that he is clean.

The thirty-sixth annual meeting of the American Veterinary Association will be held in New York City in September next. The Seventh International Veterinary Congress is to be held at Baden-Baden from August 9 to 14, 1899. The subjects to be discussed include prophylactic measures to prevent the spread of cattle-diseases by the export of animals; treatment of tuberculosis in the domestic animals; use of flesh and milk of animals affected with tuberculosis, and requirements for inspection of meat; care of foot- and mouth-diseases of swine; also care of rabies; the extension of veterinary instruction, especially by the establishment of institutions for experiments in diseases and by founding chairs of comparative medicine in veterinary colleges; the preparation of a uniform anatomical nomenclature in veterinary colleges.

There has been a notable advance made in bacteriology during the year. By means of the new method of making cultures in collodion sacs *in vivo* it has been possible for Prof. Nocard to demonstrate the identity of human and avian tuberculosis. Adopting this method, Profs. Nocard and Roux have been able to obtain pure cultures of the organisms of contagious pleuro-pneumonia in cattle, which previously had baffled all investigators. The gift of Lord Iveagh of £250,000 to the Jenner Institute for the purpose of scientific research in bacteriology and other forms of biology was a noble bequest, and will be a mighty aid in the advancement of these sciences.

The University of Pennsylvania has had an honor conferred upon it in the selection of Dr. John J. Repp for the chair of Pathology and Therapeutics in the Veterinary Department of the Agricultural College of Iowa, and also as Veterinarian to the Experiment Station. Dr. Repp goes to his new charge with a thorough equipment for the work, and, we believe, will give a good account of himself.

The inauguration of a new Governor for our State has brought to our notice the probability that in the mutations of politics Dr. Leonard Pearson might be displaced as State Veterinarian. We hope Governor Stone will see the propriety of retaining in office one so eminently qualified for the work. Dr. Pearson, in the tact displayed in the prosecution of his work and by his missionary efforts in attending the Farmers' Institutes throughout the State and imparting valuable instruction, has been able to bring the farmers of Pennsylvania into thorough sympathy with the work of the State Livestock Sanitary Board. We commend Drs. Hoskins, Ridge, Conard, and J. C. Michener also for work done in this direction.

Dr. Harger's paper on "Auto-intoxication in Carnivora," read at our last annual meeting in Pittsburg, was a valuable contribution to veterinary literature and throws much light on a hitherto obscure subject. Dr. Harger has also introduced a new operation for cribbing, which will probably be demonstrated to you at our present meeting. Dr. Merillat, of Chicago, has proposed a new mode of operation for abscess of the guttural pouches, the incision being made through the soft palate and roof of the pharynx in the median line.

We feel that we ought not to close our report without some reference to the dairy interests of our country, and in this connection we call attention to a new compilation of *National and State Dairy Laws*, by R. A. Pearson, B.S., Assistant Chief of the Dairy Division of the Department of Agriculture. The work shows the progress, present status, and future needs in this direction, and is of great interest and importance to the dairyman and veterinarian. The Secretary of Agriculture, Hon. James E. Wilson, reports that experimental exports of our best butter to Great Britain have made a very favorable impression on the market, and thinks that whenever there is a surplus of our finest butter it will find a good market in France and Germany. His recommendation that the provision of the meat-inspection law be extended to cover butter and cheese, so that these products may be branded by the United States inspectors to attest their purity, we believe to be an excellent one. The dairy products which chiefly compete with our own abroad, those from Canada and Denmark, bear such a guarantee.

The Pennsylvania State Dairy Union recently held its first annual meeting at Williamsport. The exhibition of butter and cheese held in connection with this meeting, together with dairy appliances, come as a revelation to those who have not kept in touch with the advancement in dairy operations. Dairying has now become an exact science in which the hit-or-miss operations of the past are displaced by definite processes which produce a definite result. We find that butter, like a thoroughbred dog or horse, is now judged by a scale of points as to flavor, grain, color, and salt, and the keen competition between the rival creameries necessarily insures the production of a high-grade article.

President Comfort in his address stated that there were 200,000 farmers in Pennsylvania owning a million cows, to say nothing of young stock, and these with the buildings to house them were worth at least $50,000,000. Mr. Comfort urged upon the farmers the necessity of coöperation in order to obtain equitable freight-rates and just treatment from commission merchants and middlemen. Continuing, Mr. Comfort said that "our farmers should be educated to keep cows with profit." The average for each cow in the State is 400 gallons annually. With a little more care in feeding and breeding this can be increased to 500 gallons, or $6,000,000 more in the dairymen's pockets. This can be done for the same cost with a little more education and economical management. This yield of 500 gallons is small, as many dairymen can testify, yet the increase over the present average would pay for all the fertilizers used by the farmers of the State nearly three times over. "Getting the milk there, must be the lesson of so caring for it that it shall go from the farm clean, sweet, and pure. Gaining this we can then educate the consumers to use our products in the quantities they should." Speaking of legislation as viewed from the stand-point of the farmer, Mr. Comfort says: "If the 200,000 farmers of Pennsylvania and the voters they influence were united they could have anything they asked for, provided it was just. We farmers elect the majority of the representatives; we pay a large share of the taxes; but we unitedly do nothing for the protection of our common interests. Let us unite upon measures we wish passed, and every man of us see to it that our representatives vote for them. We must watch legislation. All our oleo laws have been flung to the winds. Boards of Health ask for power to run our business, and press their measures with a zeal worthy of a better cause, and municipal authorities ask for laws concerning our business that would be considered tyrannous in a free country. Let us be just and honest in the measures we may advocate, and then if united we shall win." These words indicate that the farmers consider that they have grievances in regard to municipal legislation regulating the sale of their products. We think that in some of our large cities there ought to be a better understanding between the farmer and the local Boards of Health, and while the greatest care should be exercised to guard the health of the people, ordinances should not be passed that are onerous to the farmer and make him feel that he is the victim of a system of petty exactions.

H. B. Felton, V.M.D.,
Chairman.

